# Retrospective Descriptive Analysis of West Nile Neuroinvasive Disease (WNND) in Northwest Louisiana

**DOI:** 10.1155/2020/3513859

**Published:** 2020-05-08

**Authors:** Pradeep Kumar Mada, Philip Sneed, Gabriel Castano, Maureen Moore, Andrew Stevenson Joel Chandranesan

**Affiliations:** ^1^Infectious Diseases, Louisiana State University Health Sciences Center—Shreveport, Shreveport, LA, USA; ^2^Internal Medicine Department, Texas Health Presbyterian Hospital Dallas, Dallas, TX, USA; ^3^Internal Medicine, Louisiana State University Health Sciences Center—Shreveport, Shreveport, LA, USA; ^4^Department of Pediatrics, University of Texas, San Antonio, TX, USA

## Abstract

**Aims:**

The aim of the study was to describe the presentation characteristics and epidemiology of WNND in Louisiana to improve future recognition of cases and decrease inappropriate antibiotic use. *Settings and Design*. It was a retrospective descriptive-analytic cohort study. A total of 23 patients with WNND were identified at one tertiary care hospital center in Northwest Louisiana from a retrospective chart review from January 1, 2012 to October 31, 2017.

**Results:**

The median age was 49 years (range: 15–75) for patients with WNND. Of 23 patients diagnosed with WNND, twelve (52%) were diagnosed with encephalitis (WNE), six (26%) were diagnosed with meningitis (WNM), and five (22%) with myelitis (WNME). The common symptoms with WNND were fever in 65%, altered mental status in 61%, headache in 52%, fatigue in 43%, gastrointestinal symptoms in 43%, rigors in 30%, imbalance in 26%, rash in 9%, and seizures in 26% of patients. Most patients presented in the late summer season. The average duration of antibiotics given was six days. The average number of days from the admission to the diagnosis of WNND was nine days (3 to 16 days). Twenty-one (91%) patients survived the infection.

**Conclusions:**

Identifying WNV infection early in its clinical course would help in decreasing inappropriate antibiotic use when patients presented with fever and meningeal symptoms. Performing WNV serology in CSF studies is critical in making the diagnosis.

## 1. Introduction

West Nile virus has emerged as a major public health concern in North America causing recurring outbreaks since 1999. It is expanding its geographical range rapidly across the country and is associated with significant morbidity and mortality [1, 2]. WNV surveillance was first established in Louisiana in 2000. The virus was isolated for the first time in 2001 in Louisiana, and the worst outbreak in Louisiana occurred in 2002, resulting in 329 infections including 25 deaths [3]. The knowledge of the most common clinical presentations and epidemiology of WNND in Louisiana will guide the physicians to diagnose WNND early in its course and decrease inappropriate antibiotic use. Twenty-three patients with WNND were identified at one tertiary care hospital center in Northwest Louisiana between January 1, 2012 and October 31, 2017.

## 2. Subjects and Methods

### 2.1. Study Population

This study was approved by the Institutional Review Board of the Louisiana State University in Shreveport. A retrospective chart review was done on 23 patients with a diagnosis of WNND admitted in University Health (UH) hospital or seen in UH clinics from January 01, 2012 to October 31, 2017. We collected the data from the microbiology lab with positive WNV immunoglobulin M (IgM) or immunoglobulin G (IgG) in CSF. West Nile fever cases without neurological involvement were not included in this study.

We collected the following data: (a) demographics: age, gender, race, parish, occupation, and hobbies, (b) clinical features: presenting symptoms, physical exam findings, length of the hospital stay, and discharge condition, (c) lab parameters: complete blood count, comprehensive metabolic panel, WNV IgM in CSF, CSF glucose, protein, and cells with differentials, and (d) imaging: magnetic resonance imaging (MRI) brain and computed tomography (CT) scan of brain and spinal cord.

### 2.2. Definition of WNND

West Nile neuroinvasive disease was diagnosed based on clinical features and a positive West Nile IgM in the cerebrospinal fluid. WNND included encephalitis, meningitis, and acute flaccid paralysis.

A patient who presented with headache, neck stiffness, and/or photophobia with positive CSF WNV IgM was classified as West Nile meningitis (WNM). A patient who presented with altered mental status/confusion with positive CSF WNV IgM was classified as West Nile encephalitis (WNE). A patient who presented with acute flaccid paralysis either combined with meningoencephalitis or as an isolated myelitis, and positive CSF WNV IgM were classified as West Nile myelitis (WNME).

### 2.3. WNV Laboratory Testing

WNV serology (IgM and IgG) were performed in CSF by indirect enzyme-linked immunosorbent assay (ELISA), and results were reported as positive or negative using West Nile virus IgM/IgG capture Dx select™ by Focus Diagnostics (93% sensitive and 100% specific) [4]. Results reported as 0.89 IV or less were interpreted as negative, 0.90–1.10 IV as equivocal, and 1.11 IV or greater as positive.

### 2.4. Statistical Analysis

Statistical analysis was carried out using IBM SPSS version 20 software program (IBM^R^ SPSS^R^ Statistics, Armonk, NY). Categorical data items were summarized utilizing frequency counts and percentages, while means were calculated for age and laboratory findings. We conducted descriptive analyses to describe the study sample. Variables in the analysis included age, race, gender, comorbid conditions, and clinical features.

## 3. Results

### 3.1. Patient Geographical Data

**Figure 1:**
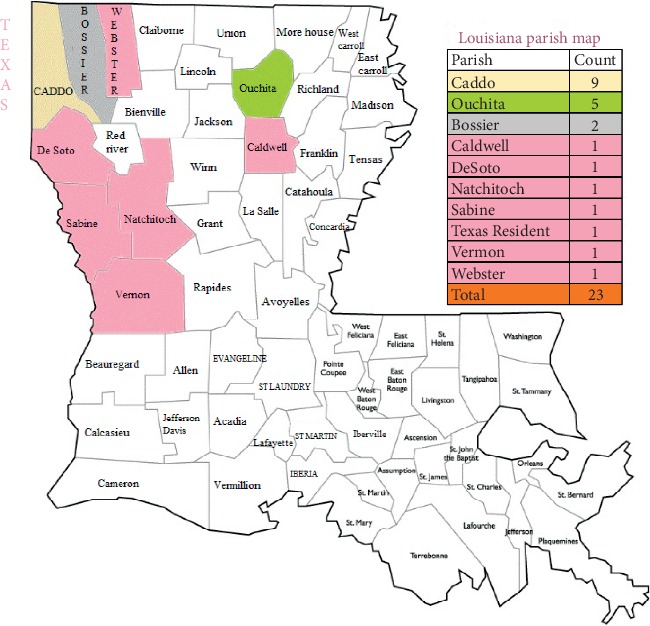
Louisiana parish map.

The Shreveport–Bossier City metropolitan statistical area is located in northwest Louisiana that includes four parishes: Caddo, Bossier, Webster, and De Soto with an estimated total population of 439,000 according to 2010 census. University Hospital is located in Shreveport, Louisiana, and it is part of the University Health System serving communities across North Louisiana, East Texas, and southwest Arkansas. A total of 23 patients with a confirmed diagnosis of WNND were found on a chart review. Out of 23 patients, nine (39%) patients were from Caddo parish, five (22%) patients from Ouachita, two (9%) patients from Bossier, and one (4%) patient each from Webster, Vernon, Sabine, Natchitoches, De Soto, and Caldwell parishes. One (4%) patient was a Texas resident (Figure 1).

### 3.2. Patient Demographics

Twenty-three patients diagnosed with WNND were included in this study. Fifteen of them (65%) were males and eight (35%) were females. The median age was 49 years (range: 15–75). Fourteen were black (61%), and nine were whites (39%).

### 3.3. Clinical Diagnosis and Preexisting Conditions

Of 23 patients diagnosed with WNND, 12 (52%) were diagnosed with encephalitis (WNE), six (26%) were diagnosed with meningitis (WNM), and five (22%) with myelitis (WNME). Nine cases of WNE were above 50 years of age. The common symptoms were fever in 65%, altered mental status in 61%, headache in 52%, fatigue in 43%, gastrointestinal symptoms in 43%, rigors in 30%, imbalance in 26%, rash in 9%, and seizures in 26% of patients (Tables 1 and 2).

Most commonly seen preexisting conditions were hypertension (74%), diabetes mellitus (35%), hyperlipidaemia (26%), coronary artery diseases (13%), human immunodeficiency virus (HIV) infection (13%), cancer (9%), and immunosuppressive drugs (4%) (Table 3).

Most patients presented in late summer; incidence was peak in the month of August with ten cases (44%) followed by July and September with four cases (17%) in each month, March with two (9%) cases, and May, February, and October with one case (4%) in each month. Only two (9%) patients recollected and gave the history of a mosquito bite.

### 3.4. WNV Testing by Serology

Both IgM and IgG were positive in the CSF of thirteen (56.5%) patients. Ten (43.5%) patients had positive IgM antibodies only against WNV, in their CSF.

### 3.5. CSF Analysis

CSF/serum glucose ratio was calculated as a surrogate for hypoglycorrhachia [5, 6]. CSF/serum glucose ratio was below or equal to 0.5 in nine (39%) patients and above 0.5 in the remaining 14 patients (61%). CSF protein was less than 45 mg/dL in two (9%) patients with WNND, greater than 45 but less than or equal to 100 mg/dL in 12 patients (52%), and greater than 100 mg/dL in nine (39%) patients. White cell count of 0–5/mm^3^ was seen in three (13%) patients, and elevated white cell count (>5/mm^3^) was present in 20 patients (87%) with WNND. Lymphocytosis (>80%) was present in seven (30%) patients with WNND (Table 4). No significant lab abnormalities were found on complete hemogram.

### 3.6. Imaging Studies

Magnetic resonance imaging (MRI) brain was performed on 14 patients, and a computerized tomographic (CT) scan of the brain was performed on 18 patients. On CT brain, no acute intracranial abnormalities were seen in 13 patients, features suggestive of microvascular disease were present in four patients, and right temporal encephalomalacia was seen in one patient. MRI brain showed various features including limbic encephalitis in one patient, extensive ischemic lesions in the right cerebral hemisphere in one patient, mild enhancement of the meninges in two patients, periventricular white matter changes consistent with microvascular disease in four patients, features consistent with meningoencephalitis in one patient, and MRI brain was unremarkable in five patients.

### 3.7. Length of Stay and Follow-Up

The average length of hospital stay for all WNND patients was 13 days, with a range from 4 to 57 days. Patients with WNE stayed an average of 13 days with a range from 2 to 57 days, whereas patients with WNM stayed an average of 13 days, with a range from 4 to 15 days, and patients diagnosed with WNME stayed an average of 11 days, with a range from 2 to 27 days. Nine (39%) patients required ICU stay (three from the WNE group, four from the WNME group, and two from the WNM group) with an average of seven days ranging from 2 to 24 days. Seven (30%) patients needed assisted ventilation (four from the WNE group and three from the WNME group) and in that, five patients were above 50 years of age.

### 3.8. Duration of Antibiotic Used before West Nile Infection Diagnosis

Only one (4%) patient was not given any antibiotics. The average duration of antibiotics was six days with a range of 0–14 days.

### 3.9. Days before Lumbar Puncture Performed

The average number of days from the admission to lumbar puncture was two days.

### 3.10. Duration of Delay in Diagnosis of WNV Infection

The average number of days from the admission to the diagnosis of West Nile infection ranged from 3 to 16 days with an average of nine days.

### 3.11. Clinical Outcome of WNV Infection after an One-Year Follow-Up

21 (91%) patients survived the infection, and two (9%) patients were deceased and both were above 50 years (one in the WNM group and one in the WNE group). Three (13%) patients were discharged to a rehabilitation center (all from the WNE group and above 50 years of age). On an one-year follow-up, eight (35%) patients had no residual deficits, four patients (three in the WNE group and one in the WNM group; surprisingly all were below 50 years of age) had residual deficits (Left leg weakness in two patients, residual headache and myoclonus in one patient, and decreased vision in one patient), and nine (39%) patients were lost to follow-up.

## 4. Study Limitations

Study limitations include a small number of patients from one hospital setting, and we used WNV IgM in CSF as a diagnosis of WNND who presented with the compatible illness. However, WNV IgM assay suffers from cross-reactivity with other flaviviruses infections. Ideally, a positive ELISA test should be confirmed by the plaque reduction neutralization test (PRNT) to exclude cross reactivity with other flaviviruses, but such testing requires biosafety level 3 facilities.

## 5. Discussion

WNND manifests as focal motor weakness, meningitis, encephalitis, an isolated myelitis, or as an overlap syndrome, and it very rarely leads to Guillain–Barré syndrome and other demyelinating neuropathies [7]. Patients with advanced age, hematologic disorders, diabetes mellitus, renal disease, alcohol abuse, or hypertension are at higher risk of developing WNND and a higher case-fatality rate. 83% of the patients had hypertension in our study population. It was previously shown in one study that hypertension was independently associated with severe West Nile illness on multivariable analysis [8]. Among WNND patients, 50–70% develop encephalitis, 15–35% develop meningitis, and 3–20% develop acute flaccid paralysis [9–16]. Our study concorded with prior studies that encephalitis was more common than meningitis with West Nile infection (12 vs 5 cases). Most WNV infections in humans are observed between July and October [17, 18]. 19 out of 23 cases in our study were presented between July and October months. WNV spreads through a vector (mosquito), and once it reaches a human host, it is a dead-end host for the virus as it does not replicate to high enough levels to sustain mosquito-borne transmission in the human body [19]. Though getting a history of mosquito bites is important, many patients could not recollect the incident. Only two patients gave the mosquito bite history in this study. Various surveillance studies found that possums, skunks, and raccoons can be carriers of WNV [20]. There is an ecological component to WNV activity, having a higher incidence in regions with forests, lakes, or rivers. There are numerous factors that contribute to viral amplification in the environment and subsequent epizootic WNV activity. Climate conditions, particularly ambient temperature and rainfall are critical drivers for mosquito abundance and amplification of WNV [21]. Though the virus is usually transmitted by mosquito bite, other modes of transmission are also reported. Two blood transfusion-related fatalities due to WNV contamination were reported in literature [22]. To reduce the risk of transfusion-transmitted infections, WNV screening for all donations has been carried out since 2003. Infected individuals should not donate blood for 120 days. AABB recommends blood centers that convert from mini-pool nucleic acid testing (MP-NAT) to the more sensitive individual donation nucleic acid testing (ID-NAT) during periods of West Nile virus activity [23]. WNV infections acquired through vertical transmission and through breastfeeding were also described in literature [24, 25].

More than 50% of patients presented with fever, altered mental status, and headache. There was an interesting male and Afro-American preponderance in our study population to develop WNND; however, the significance of this finding was unclear. There was nine days delay in the diagnosis, and antibiotics were used with an average of six days. If we diagnose early, then, we can decrease the antibiotic usage and subsequent side effects of antibiotics. More than 90% of patients survived the infection in this study. The mortality rate among WNND patients showed to be up to 20% [26, 27]. We had two deaths and both were above 50 years of age. MRI brain may show focal lesions of the thalami, basal ganglia, and spinal cord in some WNND patients; however, there were no pathognomonic features for WNND [7]. Based on electrodiagnostic tests, WNND affects the gray matter of the anterior horn cells simulating polio causing paralysis without affecting the sensory system [28]. The histopathology of WNND in other studies revealed glial nodules, mononuclear infiltration, and loss of neurons prominent in the gray matter of the medulla, pons, and midbrain. Spinal cord inflammation was universally present, predominantly in the anterior horns [29–31].

WNND is not a mandatory reportable disease by the Louisiana sanitary code; so, it is not consistently reported. CDC reported 1639 WNV infections, in which it reported 1009 WNND infections in Louisiana between 1999 and 2016. Louisiana is on the top 7 US states with an annual incidence of WNND of one per 100,000 [28]. According to the 2018 Louisiana health department surveillance report, Louisiana has had 52 WNND cases, and four deaths were reported in 2018 and the state is currently leading the country in West Nile virus cases [32]. In Caddo parish, from 2002 to 2016, there have been 131 WNV infections, been the second parish affected, next to East Baton Rouge with 139 [33]. These findings certainly grant for further research and better preventive strategies to curb WNV infection. In Louisiana, the normal course of the disease is divided into four phases, such as maintenance, amplification, epidemic, and late epidemic; it is not until the epidemic phase when we observe manifestations of neuroinvasive disease. The incidence of this disease seemed to be seasonal, having its highest peak in June to August with up to 50% of positive results for WNV [34].

Though we did not include pediatric population in our study, it is pertinent here to discuss WNV infections in children. Young children represented the majority of cases in the first recognized epidemic of WNV occurred in Israel in 1951 [35]. CDC analyzed around 400 pediatric WNND in the United States from 1999 to 2007, and it reported that the median age was 12 years, and mortality was 0.01% [36]. In previous studies conducted in USA from 1999–2012, they demonstrated that the number of WNND in pediatric patients was about 4% of the total cases of symptomatic WNV infections, making this a rare diagnosis. Only 150 out of 4000 WNV cases were of age 19 or younger in 2002 outbreak. In 2003 outbreak, 31 cases of WNV encephalitis and 79 cases of WNV meningitis occurred in pediatric population, but no fatality is reported [37, 38]. A review of WNND in 2012 in Cuyahoga County, Ohio state, has shown that children had fewer neurological symptoms, better prognosis, and lower fatality compared to adults [39]. Another study reported that 40% cases were West Nile infections with 0.25% mortality in a retrospective analysis of neuroinvasive arboviral infections in over 1200 pediatric population in 48 states collected from 2003 to 2012 [40].

A number of vaccine approaches have been explored and showed potential to combat WNV [41]. Inactivated vaccine trials in phases I and II showed 94% of effectiveness in horses [42]. A recombinant/subunit vaccine using the E. Glycoprotein showed favorable results in horses and mice as well [43].

## Figures and Tables

**Table 1:** Demographic data.

Characteristics		Total number of patients (*n* = 23)
Mean age in years	49	

Sex	Males	15 (65%)
	Females	8 (35%)

Race	Black	14 (61%)
	White	9 (39%)

Diagnosis		
Encephalitis		12 (52%)
Meningitis		6 (26%)
Myelitis		5 (22%)

**Table 2:** Clinical features of 23 patients with WNND.

	WNE (*n* = 12)	WNM (*n* = 6)	WNME (*n* = 5)	Total (%) (*n* = 23)
Fever	6 (50%)	5 (83%)	4 (80%)	15 (65%)
Rigors	2 (17%)	3 (50%)	2 (40%)	07 (30%)
Headache	1 (9%)	6 (100%)	5 (100%)	12 (52%)
Back pain	1 (9%)	2 (33%)	1 (20%)	4 (17%)
Myalgia	0 (0%)	3 (50%)	1 (20%)	4 (17%)
Fatigue	5 (46%)	3 (50%)	2 (40%)	10 (43%)
Rash	1 (8%)	0 (0%)	1 (20%)	2 (9%)
Acute flaccid paralysis	0 (0%)	0 (0%)	5 (100%)	5 (22%)
Numbness	0 (0%)	0 (0%)	1 (20%)	1 (4%)
Tingling	0 (0%)	0 (0%)	1 (20%)	1 (4%)
Imbalance	1 (9%)	2 (33%)	3 (60%)	6 (26%)
Seizures	4 (36%)	0 (0%)	2 (40%)	6 (26%)
Altered mental status	9 (82%)	0 (0%)	5 (100%)	14 (61%)
Visual complaints	0 (0%)	1 (17%)	3 (60%)	4 (17%)
Hearing complaints	1 (9%)	0 (0%)	0 (0%)	1 (4%)
Neck stiffness	0 (0%)	3 (50%)	1 (20%)	4 (17%)
Respiratory	4 (36%)	0 (0%)	2 (40%)	6 (26%)
GI symptoms	4 (36%)	3 (50%)	3 (60%)	10 (43%)
Lymph node swelling	1 (9%)	0 (0%)	0 (0%)	1 (4%)

**Table 3:** Preexisting conditions in patients with WNND.

Comorbid condition	WNE (*n* = 12)	WNM (*n* = 6)	WNME (*n* = 5)	Total (*n* = 23)
HIV	2 (17%)	0 (0%)	1 (20%)	3 (13%)
DM	3 (25%)	2 (33%)	3 (60%)	8 (35%)
HTN	10 (83%)	3 (50%)	4 (80%)	17 (74%)
Cancer	2 (17%)	0 (0%)	0 (0%)	2 (9%)
Dialysis	0 (0%)	0 (0%)	0 (0%)	0 (0%)
On immune suppressive drugs	0 (0%)	1 (17%)	0 (0%)	1 (4%)
Hyperlipidaemia	3 (25%)	1 (17%)	2 (40%)	6 (26%)
Coronary artery disease	2 (17%)	1 (17%)	0 (0%)	3 (13%)

**Table 4:** CSF analysis of patients with WNND.

CSF analysis		WNE (*n* = 12)	WNM (*n* = 6)	WNME (*n* = 5)	Total (*n* = 23)
CSF/blood glucose ratio	≤0.5	4 (33%)	3 (50%)	2 (40%)	9 (39%)
	>0.5	8 (67%)	3 (50%)	3 (60%)	14 (61%)

Protein (mg/dl)	15–45	2 (17%)	0 (0%)	0 (0%)	2 (9%)
	>45–100	5 (42%)	5 (83%)	2 (40%)	12 (52%)
	>100	5 (42%)	1 (17%)	3 (60%)	9 (39%)

WBC/mm^3^	0–5	3 (25%)	0 (0%)	0 (0%)	3 (13%)
	6–100	6 (50%)	5 (83%)	4 (80%)	15 (65%)
	101–500	3 (25%)	1 (17%)	1 (20%)	5 (22%)

Lymphocyte percentage	≤80%	7 (58%)	5 (83%)	4 (80%)	16 (70%)
	>80%	5 (42%)	1 (17%)	1 (20%)	7 (30%)

## Data Availability

The data used to support the findings of this study are included within the article.
